# *Gyrodactylus* spp. diversity in native and introduced minnow (*Phoxinus phoxinus*) populations: no support for “the enemy release” hypothesis

**DOI:** 10.1186/s13071-016-1306-y

**Published:** 2016-01-28

**Authors:** Ruben Alexander Pettersen, Kjartan Østbye, Johannes Holmen, Leif Asbjørn Vøllestad, Tor Atle Mo

**Affiliations:** Center for Ecological and Evolutionary Synthesis (CEES), Department of Biosciences, University of Oslo, P. O. Box 1066, Blindern, NO-0316, Oslo, Norway; Department of Forestry and Wildlife Management, Hedmark University College, Campus Evenstad, Elverum, NO 2418 Norway; Norwegian Veterinary Institute, P.O. Box 8156, Dep. NO-0033, Oslo, Norway

## Abstract

**Background:**

Translocation of native species and introduction of non-native species are potentially harmful to the existing biota by introducing e.g. diseases, parasites and organisms that may negatively affect the native species. The enemy release hypothesis states that parasite species will be lost from host populations when the host is introduced into new environments.

**Methods:**

We tested the enemy release hypothesis by comparing 14 native and 29 introduced minnow (*Phoxinus phoxinus*) populations in Norway with regard to the ectoparasitic *Gyrodactylus* species community and load (on caudal fin). Here, we used a nominal logistic regression on presence/absence of *Gyrodactylus* spp. and a generalized linear model on the summed number of *Gyrodactylus* spp. on infected populations, with individual minnow heterozygosity (based on 11 microsatellites) as a covariate. In addition, a sample-based rarefaction analysis was used to test if the *Gyrodactylus*-species specific load differed between native and introduced minnow populations. An analysis of molecular variance was performed to test for hierarchical population structure between the two groups and to test for signals of population bottlenecks the two-phase model in the Wilcoxon signed-rank test was used. To test for demographic population expansion events in the introduced minnow population, we used the kg-test under a stepwise mutation model.

**Results:**

The native and introduced minnow populations had similar species compositions of *Gyrodactylus*, lending no support to the enemy release hypothesis. The two minnow groups did not differ in the likelihood of being infected with *Gyrodactylus* spp. Considering only infected minnow populations it was evident that native populations had a significantly higher mean abundance of *Gyrodactylus* spp. than introduced populations. The results showed that homozygotic minnows had a higher *Gyrodactylus* spp. infection than more heterozygotic hosts. Using only infected individuals, the two minnow groups did not differ in their mean number of *Gyrodactylus* spp. However, a similar negative association between heterozygosity and abundance was observed in the native and introduced group. There was no evidence for demographic bottlenecks in the minnow populations, implying that introduced populations retained a high degree of genetic variation, indicating that the number of introduced minnows may have been large or that introductions have been happening repeatedly. This could partly explain the similar species composition of *Gyrodactylus* in the native and introduced minnow populations.

**Conclusions:**

In this study it was observed that native and introduced minnow populations did not differ in their species community of *Gyrodactylus* spp., lending no support to the enemy release hypothesis. A negative association between individual minnow host heterozygosity and the number of *Gyrodactylus* spp. was detected. Our results suggest that the enemy release hypothesis does not necessarily limit fish parasite dispersal, further emphasizing the importance of invasive fish species dispersal control.

**Electronic supplementary material:**

The online version of this article (doi:10.1186/s13071-016-1306-y) contains supplementary material, which is available to authorized users.

## Background

Anthropogenic translocation of species between ecosystems occurs worldwide at an increasing rate [1]. Some species that are introduced into new environments become invasive, imposing major negative ecological effects on the native biota [2] with concomitant economic costs for the society [3–6]. Introduced species may also act as vectors for new parasites and diseases that may infect native hosts [7]. Transmission of non-native parasite species may lead to large population-dynamic effects [8]*.* Noteworthy examples are the introduction of the monogenean *Gyrodactylus salaris* Malmberg, 1957 [9] into Norwegian rivers with subsequent dramatic decline in the native Atlantic salmon (*Salmo salar* L.) populations [10] and the crayfish plague (*Aphanomyces astaci* Schikora, 1906), a parasite that has been introduced to Norwegian watercourses and causes mass-mortalities in European crayfish (*Astacus astacus* L.) populations [11]. In these cases, strong and visible effects are evident, but often the effects of introduced parasites are difficult to observe [12].

Freshwater fish are commonly transported outside their native distribution area; this potentially leads to a loss of native fish species and that the fish communities become more similar (sometimes called species homogenization)[13, 14]. The minnow (*Phoxinus phoxinus* L.) is distributed from Urals in the east to Europe in the west. In Norway, the minnow's natural distribution is limited to the northern and southeastern parts [15]. During the last decades, new minnow populations have been established due to human activities such as fishing with live bait, stocking (intentional and non-intentional), and reorganization of waterways [16]. Genetic studies suggest that both short- and long-distance translocations of minnows have occurred between Norwegian watercourses [17, 18]. Several species of ectoparasites of the genera *Gyrodactylus* have been reported from minnow in Europe [9, 19, 20]. The diversity of *Gyrodactylus* spp. on Norwegian minnow is not well known, but earlier studies indicate that up to five *Gyrodactylus* species can be found [21]. The *Gyrodactylus* fauna in Norway is depauperate compared to the rest of Europe [22]. *Gyrodactylus* spp. often exhibit a high degree of host-specificity and a direct life cycle where transmission typically takes place after direct contact with a new host [23]. These traits make *Gyrodactylus* spp. particularly tractable for parasite studies, as it is not necessary to take infracommunities from intermediate hosts into consideration [24]. The effects of *Gyrodactylus* spp. on minnow hosts are not known, but based on other *Gyrodactylus* - host systems it is reasonable to assume they impose negative fitness impacts [25].

The “enemy release hypothesis” (ERH) states that introduced species lose some of their natural enemies such as pathogens and parasites in the new environment [26, 27]. This will provide a fitness advantage as less energy is used to respond to the parasites and more can be allocated to growth and reproduction. Comparing native and introduced plant species, Mitchell and Power [28] found that introduced plants harboured less fungi and virus species than plants in their native habitats. Further, Torchin et al. [29] compared 26 host taxa (molluscs, crustaceans, fishes, birds, mammals, amphibians and reptiles) and showed that introduced species had half the number of species of parasites compared to the native species. A comparison of 176 different studies addressing the enemy release hypothesis found almost as many studies in support (36 %) as questioning the hypothesis (43 %) [30]. There is also some support for the ERH-hypothesis in freshwater fish [31–34]. However, few studies have addressed this topic at the population level. Halvorsen [35] hypothesized that local movement of fish between neighbouring water bodies would similarly disseminate their parasites, assuming that ecological conditions were similar. Under this hypothesis, the prediction would be that transport of individuals between closely located water bodies would lead to a more similar parasite fauna in native and introduced hosts than if hosts are introduced to more geographically distant locations. Alternatively, the number of parasites might increase in introduced populations compared to native populations by acquiring new parasites in the new environments from resident hosts [36, 37].

There are several factors that can affect the abundance of and resistance to parasites in host species, both in native and introduced populations. One such factor is heterozygosity, measured by neutral genetic markers, which is hypothesized to be associated with fitness (review by Chapman et al. [38]), and fitness-related traits such as resistance to parasite infections [39]. The assumption is that a genetically diverse host has a more robust immune system to handle parasite infections as it holds a larger diversity of anti-parasite specific genes [40, 41]. Several studies on fish species have documented that selection acts on MHC genes related to e.g. monogenean infection (reviewed by Alvarez-Pellitero, [42]). Also, demographic bottlenecks during introduction to the new environments may result in reduced genetic diversity in host and/or parasite population, putatively affecting persistence and fitness in the new environment [43].

The main aim of this study was to test the enemy release hypothesis using a dataset on native and introduced minnow populations in Norway. First we tested if the diversity of *Gyrodactylus* species differed between native and introduced minnow populations. Then we tested for variation in prevalence (i.e. presence and absence) and intensity of *Gyrodactylus* (species pooled) between native and introduced minnow. By estimating multilocus heterozygosity of the minnow hosts using a set of eleven neutral microsatellites we tested for association between individual heterozygosity and intensity. We also tested if the transplanted minnow populations had gone through demographic bottlenecks and subsequent population expansions, which potentially could explain some of our results.

## Methods

### Study area and sampling

**Fig. 1:**
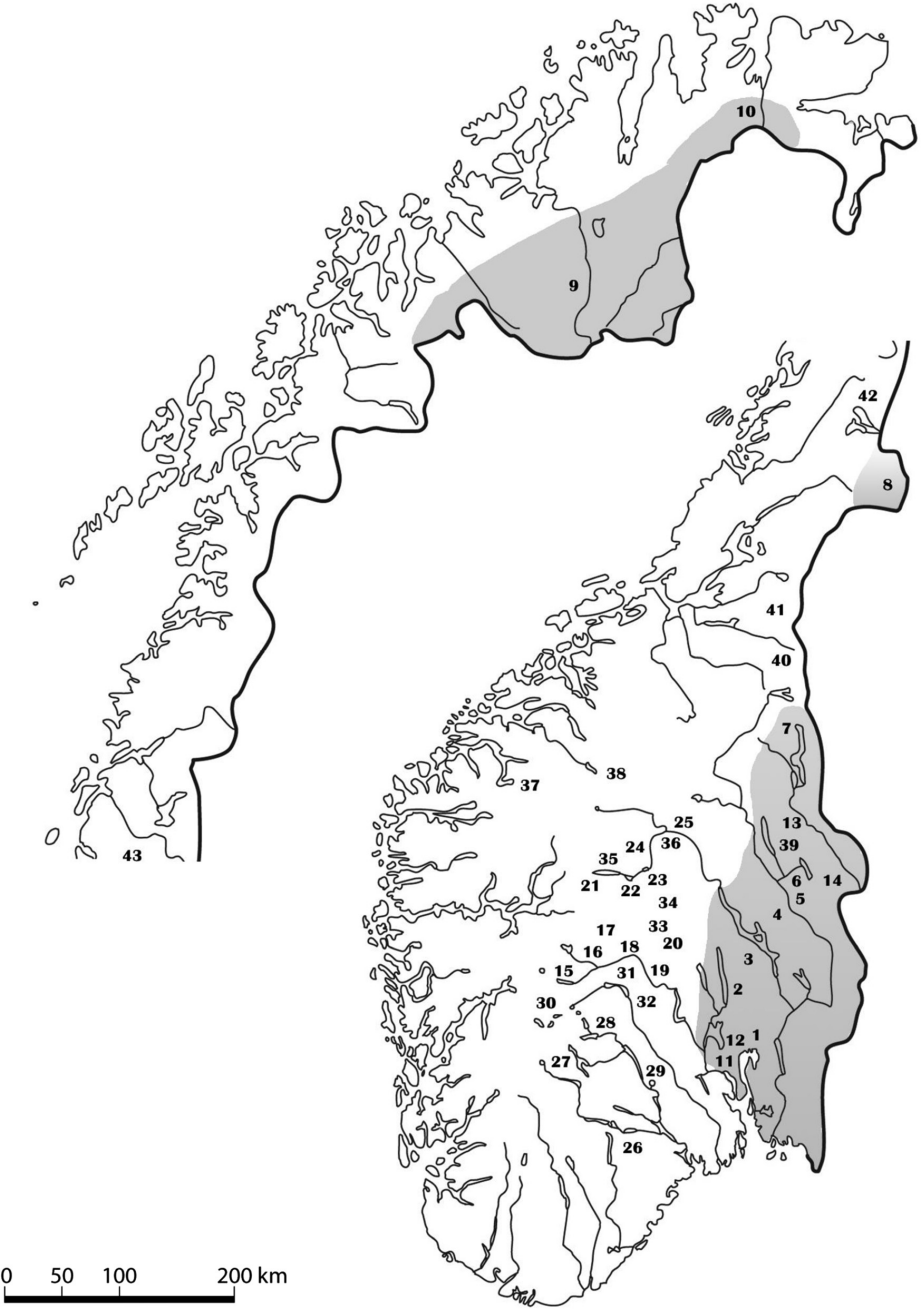
Sampling locations in Norway. The 43 minnow sampling locations in Norway (see Table 1). The 11 native minnow populations were collected from the grey part of the map

The sampling sites were selected to cover most of the distribution of native [15] and introduced [17, 44] minnow populations in Norway (Fig. 1, Table 1). The minnow populations, both native and introduced, are localized geographically far apart, usually in different watersheds. It is thus highly unlikely that there is or has been natural dispersal between the different minnow populations. The native minnow populations were all found below the upper marine limit, this limit is regarded as limiting the dispersal of minnow. The introduced minnow populations were mainly located in mountainous regions in southern Norway, and all above the upper marine limit [16]. Potentially, non-native minnow could have been introduced into native populations, leading to native and non-native minnow living in sympatry. However, a detailed study of the genetic population structure of minnow in many of these lakes indicated that this was not the case [17]. The Norwegian freshwater fish fauna is depauperate and in most populations only one or two species are present. Details on the composition of fish species at the different sampling locations is presented in Additional file 1. Brown trout (*Salmo trutta)* was the only species present at all sampling sites.

**Table 1:** Summary information for the sampled minnow *Phoxinus phoxinus* populations (11 native and 29 introduced; location number refers to Fig. 1). Location name, river name, sample size of minnow, number of minnows infected with *Gyrodactylus* spp., and infection intensity (summed number of *Gyrodactylus* spp.) in the population are given. Three measures of mean population specific genetic variability are also given: heterozygosity, gene diversity and allelic richness (with standard deviations)

Location number	Location	River	N fish	Infected fish	*Gyrodactylus* ssp*. s*ummed individuals	Heterozygosity (SD)	Gene diversity (SD)	Allelic richness (SD)
	Name							
Native								
1*	Sørkedalselva	Lysaker	55	17	41	0.514 (0.115)	0.527 (0.370)	1.527 (0.355)
2*	Fallselva	Drammen	41	9	14	0.603 (0.093)	0.567 (0.386)	1.567 (0.356)
3*	Hunnselva	Glomma	45	11	19	0.578 (0.112)	0.585 (0.340)	1.585 (0.294)
4*	Elverum	Glomma	18	13	27	0.605 (0.156)	0.675 (0.356)	1.673 (0.291)
5*	Julussa	Glomma	10	10	28	0.532 (0.107)	0.604 (0.362)	1.600 (0.321)
6*	Søre Osa	Glomma	20	10	12	0.564 (0.132)	0.598 (0.356)	1.596 (0.312)
7*	Femunden	Trysil	22	8	11	0.536 (0.111)	0.618 (0.343)	1.616 (0.289)
8*	Sørli	Sørli	19	10	16	0.472 (0.103)	0.500 (0.351)	1.499 (0.327)
9*	Stuorajavri	Alta	21	10	35	0.621 (0.186)	0.592 (0.371)	1.590 (0.348)
10*	Tana	Tana	25	6	7	0.579 (0.161)	0.594 (0.348)	1.594 (0.302)
11	Asdøltjern	Lier	40	0	0	0.509 (0.120)	0.533 (0.369)	1.532 (0.342)
12	Sagelva	Åros	54	1	1	0.474 (0.114)	0.481 (0.369)	1.481 (0.351)
13	Fiskebekktjern	Trysil	20	0	0	0.525 (0.178)	0.582 (0.344)	1.580 (0.301)
14	Landsjøen	Trysil	17	0	0	0.503 (0.148)	0.564 (0.338)	1.561 (0.297)
Introduced								
15*	Ørteren	Hallingdal	20	5	11	0.522 (0.092)	0.544 (0.367)	1.543 (0.336)
16*	Strandavatn	Hallingdal	33	14	32	0.575 (0.119)	0.566 (0.341)	1.566 (0.300)
17*	Stolsvatnet	Hallingdal	60	35	129	0.526 (0.118)	0.582 (0.330)	1.581 (0.282)
18*	Hustjern	Hallingdal	16	5	6	0.514 (0.123)	0.485 (0.355)	1.486 (0.334)
19*	Hallingsdalselva	Hallingdal	22	10	18	0.517 (0.090)	0.560 (0.379)	1.559 (0.348)
20*	Tisleia	Begna	40	15	36	0.567 (0.113)	0.577 (0.300)	1.578 (0.243)
21*	Bygdin	Vinstra	36	5	6	0.468 (0.120)	0.484 (0.340)	1.484 (0.315)
22*	Vinstri	Vinstra	28	18	20	0.545 (0.113)	0.576 (0.290)	1.575 (0.231)
23*	Vinstervatna Ø	Vinstra	122	11	14	0.599 (0.129)	0.606 (0.294)	1.606 (0.232)
24*	Birisjøen	Sjoa	21	10	12	0.632 (0.098)	0.555 (0.315)	1.557 (0.270)
25*	Otta	Otta	8	8	21	0.477 (0.118)	0.534 (0.371)	1.530 (0.342)
26	Mjåvatn	Tovdal	18	0	0	0.495 (0.148)	0.516 (0.332)	1.515 (0.299)
27	Totak	Skien	20	1	1	0.514 (0.090)	0.499 (0.385)	1.500 (0.367)
28	Møsvatn	Skien	17	0	0	0.381 (0.134)	0.489 (0.332)	1.477 (0.295)
20	Follsjå	Skien	15	0	0	0.409 (0.163)	0.453 (0.367)	1.449 (0.351)
30	Stigstuv	Numedal	52	0	0	0.515 (0.117)	0.536 (0.333)	1.536 (0.296)
31	Lægreid	Hallingdal	54	0	0	0.549 (0.111)	0.576 (0.346)	1.576 (0.303)
32	Tunhovd	Numedal	33	1	1	0.529 (0.098)	0.536 (0.379)	1.536 (0.353)
33	Kippesjøen	Etna	17	0	0	0.526 (0.134)	0.509 (0.357)	1.509 (0.343)
34	Heggefjorden	Begna	25	4	4	0.559 (0.135)	0.563 (0.338)	1.564 (0.297)
35	Vinstrervana V	Vinstra	44	0	0	0.545 (0.117)	0.586 (0.292)	1.585 (0.230)
36	Grovi	Otta	20	1	1	0.464 (0.065)	0.496 (0.418)	1.495 (0.405)
37	Jølstervatn	Jølster	38	0	0	0.501 (0.102)	0.505 (0.373)	1.505 (0.352)
38	Lesjaskogsvatn	Gubransdal	16	2	2	0.577 (0.102)	0.580 (0.323)	1.579 (0.273)
39	Glasåtjhern	Glomma	10	0	0	0.677 (0.115)	0.620 (0.365)	1.619 (0.315)
40	Essandsjøen	Nea	30	3	3	0.441 (0.128)	0.540 (0.391)	1.538 (0.365)
41	Risvatnet	Inna	20	0	0	0.409 (0.099)	0.442 (0.396)	1.441 (0.387)
42	Limingen	Namsen	20	0	0	0.595 (0.095)	0.586 (0.339)	1.586 (0.294)
43	Store Majavatn	Vefsna	16	0	0	0.419 (0.061)	0.421 (0.393)	1.422 (0.388)

*Samples used in the rarefaction analysis using EstimateS 8.2.0 (Colwell 2011) where more than 5 *Gyrodactylus* spp. individuals were observed in the population. This number of observed *Gyrodactylus* spp. individuals is needed to calculate rarefaction using the software EstimateS 8.2.0.

The water temperature regimes may differ between native minnow populations located at lower altitudes below the upper marine limit compared with introduced minnow usually found in high mountain lakes. Based on recent genetic studies, the newly founded minnow populations seem to have a diverse origin, some originating from fish transported at local scales and some transported at regional (European) scales [17, 18].

A total of 1278 minnow were randomly sampled from 43 populations (14 native; 29 introduced) during the years of 1997–2003, mainly during August and September (Fig. 1, Table 1). The introduced populations were identified based on their elevation above sea level (i.e. being situated outside the main natural distribution area of the minnow in Norway) as well as not having prior records of minnow occurrence [15, 44, 45]. However, the original sources of the introduced minnow populations are unknown, precluding a direct comparison between introduced and source populations.

Minnows were captured in small rivers/streams and in lakes close to the shoreline, using backpack electric fishing equipment. After capture, all minnows were immediately euthanized and stored on 96 % ethanol for later analysis. However, in some cases only the caudal fin was preserved in ethanol for later analysis (see description below). There is no information available on the density of minnow in the different lakes.

### *Gyrodactylus* spp. identification

In the laboratory, the caudal fin was excised from each fish that had been preserved as a total individual. These excised caudal fins and the caudal fins that were excised and conserved in the field were then investigated in detail. The caudal fin was chosen in order to utilize the total material of minnow samples. Thus we here assume that parasite counts on the caudal fin are representative for the total *Gyrodactylus* spp. community. To assess this approach previously unpublished data (Pettersen, R.A.) were used to compare the number of *G. aphyae*, *G. magnificus*, and *G. macronychus* on the caudal fin with the number of specimen on the total body (using protocol developed by Buchmann, [46]). The results showed that the correlation was strong (Spearman *ρ* = 0.871, *p* = 0.0001. Nfish = 73).

Each fin was inspected using a stereomicroscope (40X) to count the number of *Gyrodactylus* spp. individuals. All *Gyrodactylus* spp. individuals were carefully removed for preparation for species identification using light microscopy. However, three individuals were lost during the preparation procedures.

Each *Gyrodactylus* individual was identified to species based on the haptoral hard parts [47]. The haptoral hard parts were digested with Proteinase K (1 % in buffer) until all the tissue was removed, and were mounted on a microscope slide in formaldehyde-glycerine (15:85) fixative. A Leica DM 4000 B microscope with a Heine phase contrast condenser, and a 100X/1.25 oil immersion objective (no. 506159) was used. This equipment was linked to a Leica DFC 320 digital camera and archiving system, and the Leica software LAS_©_ to take pictures. These pictures were used to identify the different *Gyrodactylus* species. To establish the identity of the different *Gyrodactylus* species we used information from the first description and follow-up descriptive literature [9, 48]. We also used an additional visual identification as recommended by Shinn et al. [49] comparing our samples with pictures from the database GyroDb [22]. Five species of *Gyrodactylus* have previously been reported on minnow from Norway: *G. laevis*, Malmberg 1957, *G. magnificus*, Malmberg 1957, *G. phoxini*, Malmberg 1957, *G. macronychus*, Malmberg 1957, *G. aphyae*, Malmberg 1957 [50]. A further nine *Gyrodactylus* species are reported on minnow elsewhere in Eurasia [22]. One of the authors (Pettersen, R. A.) first identified all the *Gyrodactylus* species and counted all individuals, while another (Mo, T. A.) checked and confirmed all the species identifications.

### Genetic diversity in the minnow hosts

Neutral multilocus genetic diversity for the minnow hosts was assessed by genotyping 11 microsatellites for a total of 1278 minnows. The 11 microsatellites were selected from a set of 36 microsatellite markers developed for cyprinids (the markers codes are: Z7634, Ca1, Ca3, Ca5, Ca6, Ca12, MFW1, MFW17, GF11, Z15751, Z9692) [17, 51]. Here, individual and population level heterozygosity were estimated using GenAlEx 6.5 [52], while gene diversity and allelic richness was estimated for populations using Fstat 2.9.3.2. [53]. Population estimates of these three measures of genetic diversity are given in Table 1. We used estimated hetereozygosity as a covariate in the statistical analyses, as Chapman et al., [38] suggests that this is a robust measure of genetic diversity. It is also assumed that heterozygosity is positively associated with parasite resistance (e.g. Blanchet et al., [39]).

To test for hierarchical population structure between native and introduced minnows, as well as for a geographical pattern of population subdivision, analysis of molecular variance (MANOVA, [54]) in the program GenAlex 6.5 was used [55]. Genetic variance (based on ϕ_PT_) was partitioned among minnow individuals within populations, among populations, and among the population types (native and introduced minnows) using 9999 permutations.

If heterozygosity is important for individual parasite resistance in minnow hosts, demographic population bottlenecks associated with transfer of minnow to new locations may be important. To test for heterozygosity excess, being a signal of a population bottleneck, the Bottleneck 1.2.0.2 software was used [56]. Here, results were evaluated based on the two-phase model (TPM) in the Wilcoxon sign-rank test (1000 iterations), and the "mode - shift" indicator, which discriminates bottlenecked populations from demographically stable populations [57].

After introduction of minnows to new locations a demographic population expansion may occur, which can potentially be linked to persistence against parasites. To test for a demographic population expansion event in the introduced minnow population, we used the kg-test under a stepwise mutation model (SMM) [58]. Here, the k-test compares intra-locus allelic distributions between expanding and stable populations, while the interlocus g-test compares variance in number of repeats between expanding and stable population [58, 59]. A significant number of negative k-values indicate a signature of a demographic population expansion. The g-test significance level was compared to the recommended cut-off value in Table 1 (p. 455) in Reich et al., [58].

### Statistical analyses

In a number of populations we did not find any individual minnows infected with *Gyrodactylus* spp. To test if the probability of being infected with *Gyrodactylus* spp. differed between native and introduced minnow populations, we used a nominal logistic regression (binomial distribution, logit link, the distribution of *Gyrodactylus* spp. is given in Online Resource 1). Presence or absence of *Gyrodactylus* spp. was used as the response variable, population group (native or introduced) as factor, and average heterozygosity as a covariate. Here, all *Gyrodactylus* species were pooled (43 minnow populations; 1278 fish individuals).

Further, *Gyrodactylus* species accumulation curves were calculated for each of the native and introduced minnow groups using EstimateS 8.2.0 [60]. This is a sample-based rarefaction curve that gives species accumulation as a function of occurrence, presenting associated 95 % confidence intervals. These curves allow species richness comparisons among test groups, while accounting for differences between locations in the number of individual minnows sampled [61, 62]. Here, only 10 native and 11 introduced populations out of the original 44 populations (Table 1) were used since the sample size of *Gyrodactylus* specimens in each population must be larger than the total number of *Gyrodactylus* species observed in the total dataset [60]. Thus, in all the 21 selected minnow populations more than four *Gyrodactylus* specimens was observed.

The mean number of *Gyrodactylus* specimens per host was estimated for native and introduced minnow populations, using the minnow populations where *Gyrodactylus* spp. were observed (29 populations, 880 individuals). We used a generalized linear model with the number of *Gyrodactylus* specimens as a response variable (using a Poisson error distribution and a log-link, using a maximum likelihood estimation method), and native or introduced minnow populations, population identity nested under the two groups, heterozygosity, and interaction between heterozygosity and native or introduced population groups as factors (interaction was not significant and thus removed from further analyses).

The same statistical test was applied but only using minnow hosts that were infected comprising 21 populations and 253 minnow hosts. Here, again the interaction between heterozygosity and the native or introduced population groups as factors was not significant and subsequently removed from the analyses.

All the statistical analyses, except the rarefaction analyses, were implemented in JMP 9.0 (SAS, 2012) [63].

## Results

### *Gyrodactylus* species occurrence

A total of 515 *Gyrodactylus* specimens were examined and identified. Four species were found: *G. magnificus*, *G. phoxini*, *G. macronychus*, and *G. aphyae* (Table 2). *G. aphyae* was the most common species being present in all the 21 infected minnow populations. *G. magnificus* was found in 12 populations, *G. macronychus* in 10 populations. *G. phoxini* was only found in 3 minnow populations. Most commonly, only one species of *Gyrodactylus* was found on each minnow, rarely two species of *Gyrodactylus* were found on the same tail fin. The combination of *G. aphyae* and *G. magnificus* was the most common, being detected on 11 minnows (from 10 populations). The combination of *G. aphyae* and *G. phoxini* was detected on 3 minnows (from 3 populations), and one minnow had the combination of *G. magnificus*, and *G. macronychus.*

**Table 2:** The number of minnows from native and introduced populations infected with *G. aphyae*, *G. macronycus*, *G. magnificus*, *G. phoxini*. For population numbers se Fig. 1

Population number	Location		Number of *minnows* infected with			
		*N fish*	*G. aphyae*	*G. macronycus*	*G. magnificus*	*G. phoxini*
*Native populations*						
1	Sørkedalselva	55	9	5	6	-
2	Fallselva	41	9	-	-	-
3	Hunnselva	45	8	5	-	-
4	Elverum	18	13	-	-	-
5	Julussa	10	2	1	10	-
6	Søre Osa	20	1	2	7	-
7	Femunden	22	4	5	-	-
8	Sørli	19	6	3	3	-
9	Stuorajavri	21	3	-	1	8
10	Tana	25	1	5	-	-
*Introduced populations*						
16	Ørteren	20	5	-	-	1
17	Strandavatn	33	1	-	14	-
18	Stolsvatnet	60	35	2	5	2
19	Hustjern	16	5	-	-	-
20	Hallingdalselva	22	1	-	9	-
21	Tisleia	40	3	-	12	-
22	Bygdin	36	2	-	4	-
23	Vinstri	28	11	7	1	-
24	Vinstervanta Ø	122	5	6	2	-
25	Birisjøen	21	10	-	-	-
26	Otta	8	8	-	-	-

### Genetic variation and demographic tests in minnow populations

A total of 1278 minnows were genotyped for 11 microsatellites, and the observed individual level of heterozygosity ranged between 0.091 and 1.0, with the overall mean 0.534 for all fish. The population level heterozygosity (N = 43 populations) ranged from 0.381 (Møsvatn) to 0.632 (Birisjøen). Gene diversity ranged from 0.421 to 0.675, and allelic richness ranged from 1.422 to 1.673 (Table 1). Based on the MANOVA analysis the genetic variance was partitioned with 77.7 % among individuals within the population level, 21.7 % at the population level and 0.6 % at the among group level (native and introduced) (Table 3). None of the populations showed a significant signal of a bottleneck event (The results are given in Additional file 2). Based on the population expansion analyses (k and g-tests), there were no significant signals of demographic expansion in any of the sampled populations (Additional file 2).

**Table 3:** Analysis of molecular variance (AMOVA) based on 11 microsatellites for the 43 minnow populations. The two groups are defined by 29 introduced and 14 native minnow populations. Variance is partitioned among the groups, among populations and within populations using random permutations

Variance component	Sum of squares	Degrees of freedom	% total	Variance	*P* value
Among groups	116.71	1	0.55	0.06	<0.0001
Among population	2713.86	41	21.70	2.02	<0.0001
Within population	8937.14	1235	77.71	7.24	<0.0001
Total	11767.74	1277		9.31	<0.0001

### Gyrodactylus species diversity and prevalence

Out of the 43 surveyed populations, 15 minnow populations were not infected with *Gyrodactylus* spp. (3 native and 12 introduced), while 28 minnow populations had at least one or more hosts infected by one or more *Gyrodactylus* spp. specimens. A total of 253 individuals in the samples were infected with at least one *Gyrodactylus* sp. The overall prevalence (i.e. the proportion of individual minnows infected with one or more *Gyrodactylus* spp. in a given population) ranged between 0 and 100 %. The prevalence did not differ between native and introduced minnows (χ^2^_1_ = 4.252, *P* = 0.119), and there was no effect of average heterozygosity (χ^2^_42_ = 2.518, *P* = 0.113).

The number of *Gyrodactylus* species did not differ on minnows classified as belonging to either native or introduced populations (tested using EstimateS; χ^2^_1_ = 0.029, *P* = 0.865). The diversity varied from 1 to 4 *Gyrodactylus* species per populations, and the predicted mean number of *Gyrodactylus* species was 4 species both in the native (95 % confidence interval: 3.9-4.1) and the introduced populations (3.8-4.2).

The intensity of *Gyrodactylus* spp. per individual minnow host ranged between 1 and 19 (see Additional file 3). When using all the 29 infected populations (880 minnows in total) it was found that the mean number of *Gyrodactylus* individuals (all species) per host differed significantly between the native (0.64 ± 1.34, mean ± SD) and introduced (0.35 ± 1.58) minnow groups (whole model: χ^2^_28_ = 553.4, *P* < 0.0001), where the introduced group had a lower infection (Table 4). The abundance was significantly negatively associated with individual heterozygosity, and there was no interaction effect (Table 4).

**Table 4:** Summary result from the generalized linear model on number of *Gyrodactylus* spp. Individuals per minnow host, with population group (native or introduced minnow populations) as factor and individual minnow heterozygosity as covariate. Population identity was nested under population group. In this test we only used the infected populations (29 populations, 880 individuals)

Factores	Sum of squares	Degrees of freedom	*p*
Among groups	8.84	1	0.003
Populations nested in groups	545.15	26	<0.0001
Heterozygosity	10.69	1	0.001

## Discussion

The “enemy release hypothesis” suggests that introduced species should harbour fewer parasite species than native species. The observed results do not support the enemy release hypothesis as similar numbers of *Gyrodactylus* species were observed on native and introduced minnow hosts, and they had the same likelihood of being infected with *Gyrodactylus* spp. In support of the observed results, Daverdin [64] compared some native minnow populations to an introduced minnow population (one of the lakes in our study) and observed no difference in the internal parasite fauna showing that the same fauna was established in the new environment. The same result was found in a study of the common carp (*Cyprinus carpio* L.) where there were no differences in helminth communities between native and introduced populations [65]. Several other studies have found some support for the enemy release hypothesis spanning a range of organisms including freshwater fish [29, 31, 33, 66, 67]. However, the success of parasite species introductions will likely depend on the complexity of the parasite lifecycle. Parasites with a lifecycle that requires more than one host will likely have a lower probability of introduction and establishment in a new environment [68] than parasites with a direct life-cycle (no intermediate hosts).

An alternative to the enemy release hypothesis is that the number of *Gyrodactylus* species increase in the introduced populations due to transmission of new *Gyrodactylus* species from other fish species already present in the new environment. However, this seems unlikely in the case of the minnow as most *Gyrodactylus* species seem to be host specific [22]. Further, it is possible that abiotic environmental conditions are also important factors determining species numbers [35]. Thus, if the environmental conditions differ strongly also the parasite fauna may differ. Environmental conditions are usually more similar for geographically close locations. Also, multiple introductions from the same source population would likely ensure that the whole parasite species fauna would be found in both environments. Thaulow et al. [18] have shown that multiple introductions of minnows have occurred from different sources into one of the same river systems studied here (River Skiensvassdraget). Thus, it is possible that multiple introduction events of minnows into lakes in this study could partly explain the similarity of the *Gyrodactylus* species fauna we observed in native and introduced minnows.

The abundance of *Gyrodactylus* was observed to be lower in introduced minnow compared to native populations in this study. This seems at odds with the enemy-release hypothesis. However, the enemy-release-hypothesis may be imprecise as it usually only considers presence or absence of parasite species. The parasite species-specific abundance of hosts is not taken into account. It is reasonable to assume that the more diverse parasite species infection a host has, as well as the abundance of each species, the higher challenge will be imposed on the immune system of the host [69]. Torchin et al. [29], who studied introduced and native populations of a set of diverse organisms observed that the mean number of parasite individuals within parasite species were lower in introduced than in native populations, similar to our observation of *Gyrodactylus* on the minnows.

Most of the studies that test the enemy release hypothesis do not have data on individual heterozygosity. In this study, we found no significant association between mean heterozygosity and the probability of being infected with *Gyrodactylus* (absence versus presence of infection) when using the whole dataset. However, when using only infected minnow there was a significant negative association between individual heterozygosity and mean number of *Gyrodactylus*. Here, the association of a higher *Gyrodactylus* infection level in more homozygotic minnow hosts was found for both native and introduced populations. This may indicate that more diverse hosts are better able to combat the infections. In two studies on the rostrum dace (*Leuciscus leuciscus* L.), Blanchet et al., [39, 70] tested if heterozygosity was associated with the mean number of the harmful fin-feeder ectoparasite *Tracheliastes polycolpus* Nordmann, 1832. They observed that parasite burdens were highest in hosts being moderately heterozygous, while extremely homozygous and heterozygous hosts had a lower parasite burden. This result seems in conflict with our observations for the minnow*-Gyrodactylus* system. However, this apparent conflict may be caused by different ranges of genetic variation among hosts in the various studies*.*

It is likely that the number of founder populations differ between native and introduced minnow populations. Also, the degree of heterozygosity may be associated with the success of founder populations [71]. However, the lack of significant bottleneck signals in the minnow populations suggested that no drastic decrease in genetic diversity occurred during colonization events (although the power of the test may be weak; see [57, 72]). This is also supported by the results from the AMOVA analysis, showing that only 0.6 % of the genetic variation was partitioned between the native and introduced populations. Thus, the relatively high level of genetic variation in the introduced populations could help explain that the two groups had the same number of *Gyrodactylus* species observed (from species accumulation curves)*.* This evaluation is valid under the assumption that similar levels of heterozygosity reflects similar abilities to withstand negative impacts from *Gyrodactylus* spp. infection, and that heterozygosity based on neutral microsatellites is correlated to genetic variation in e.g. adaptive immunocompetence genes (MHC) associated to parasite resistance [40, 73].

### Geographical distribution of *Gyrodactylus* spp. on minnows

In order to place the findings of this study in a biogeographical framework we here report on the distribution of *Gyrodactylus* spp. in Norway and other parts of Europe. A total of fourteen species of *Gyrodactylus* have been described on minnow on a global scale [22]. In this study, a total of four out of five previously reported *Gyrodactylus* species in Norway [50] were observed in the 43 minnow populations. To the southeast, in Sweden, two more *Gyrodactylus* species have been found on minnow [9]. The most plausible explanation for why Norway has a lower number of *Gyrodactylus* species than the rest of Europe is Norway`s relatively recent deglaciation (<10 000 years ago) and location to the west on the Scandinavian peninsula, with relatively long colonization routes from assumed glacial refugia. For minnows these refugia are probably situated in south central Europe (based on species determination of dated bones from the Eem interglacial (ca .150 000 years before present) [74], and likely also somewhere in Russia. Indeed, hosts at the geographical limits of their distribution often have fewer parasites in general or lack species-specific parasites (see [75]). In this study, a maximum of four *Gyrodactylus* species was observed in a minnow population. This finding is not too different from other European studies, in which a maximum of six *Gyrodactylus* species have been found in a single minnow population [19, 20]. In the current study, *G. aphyae* was found in all populations, while the three other species were more or less rare. *G. phoxini* was found in only three locations, detected on a few hosts only. The two most common species (*G. aphyae* and *G. macronychus*) in this study were also the most common species reported in the literature on *Gyrodactylus* on European minnow [19, 20]. Most commonly, only one *Gyrodactylus* species, rarely two, was observed on the individual hosts. If two species co-occurred, the combination of *G. aphyae* and *G. magnificus* was the most commonly observed.

The environmental conditions in a given lake may likely affect the establishment of the hosts in the new environments, as well as being important for survival and demographics of *Gyrodactylus* on hosts [76–79]. In this study, this could influence the mean number of *Gyrodactylus* as lakes are situated at different altitudes and thus comprise a range of environmental regimes for *Gyrodactylus* spp. Further, introduced minnow populations may need to be of a certain size in order to uphold a viable population of *Gyrodactylus* [80]. Also the population density and behaviour of minnows in a new location may be important with regard to horizontal transmission and population dynamics of *Gyrodactylus* [81].

Physio-chemical conditions of the lake environment could affect the success of establishment of minnows and its parasite fauna during introduction to new environments. Such factors could be e.g. pH and water temperature as these factors have been shown to be associated with *Gyrodactylus* spp. development and survival [77–79]. In our study, this could influence the results as lakes are situated at different altitudes and thus comprise differential temperature regimes for *Gyrodactylus* spp. However, the wide geographic range covered by both native and non-native populations suggests that such abiotic drivers do not significantly bias the results.

## Conclusions

In this study it was observed that native and introduced minnow populations did not differ in their species community of *Gyrodactylus* spp., which lends no support to the enemy release hypothesis. However, the average number of parasites per host was higher in the native than in the introduced minnow. Interestingly, a negative association between individual minnow host heterozygosity and abundance was detected*,* being evident in both the native and introduced minnow populations. Our results suggest that the enemy release hypothesis does not necessarily limit fish parasite dispersal, further emphasizing the importance of invasive fish species dispersal control.

## Additional files

Additional file 1: Table S1. A table of other fish species in the sampled localities. All the Norwegian minnow (*Phoxinus phoxinus*) populations used in the study and the other fish species in the same locality. The species of fish are: AC = Arctic charr (*Salvelinus alpinus*) BL = bleak (*Alburnus alburnus*), BR = bream (*Abramis brama*), BT = brown trout (*Salmo trutta*), BU = burbot (*Lota lota*), CC = crucian carp (*Carassius carassius*), CH = chub (*Leuciscus cephalus*), EE = eel (*Anguilla anguilla*), GR = grayling (*Thymallus thymallus*), PE = perch (*Perca fluviatilis*), PI = pike (*Esox lucius*), RU = ruffe (*Gymnocephalus cernuus*), SS = Siberian sculpin (*Cottus poecilopus*), VE = vendace (*Coregonus albula*), WB = white bream (*Blicca bjoerkna*), WF = European whitefish, SM = smelt (*Osmerus eperlanus*), ID = ide (*Leuciscus idus*), DA = dace (*Leuciscus leuciscus*), 9-SB = nine-spined stickleback (*Pungitius pungitius*) and 3-SB = tree-spined stickleback (*Gasterosteus aculeatus*). (DOC 192 kb) Additional file 2:
**A table of tests result of demographic bottleneck.** All the Norwegian minnow (*Phoxinus phoxinus*) populations used in the study. Information from tests of demographic bottleneck using Bottleneck 1.2.0.2. (Piry et al. 1999) with the two-phase model (TPM) and the Wilcoxon sign-rank test, and demographic expansion (where the intra-locus k-test identify signals of recent population expansion, while the inter-locus g-test identify signals of more ancient population expansion) using Kg-test (Reich et al. 1999). Footnotes: *Samples used in the rarefaction analysis using EstimateS 8.2.0 (Colwell 2011) where more than 5 *Gyrodactylus* spp. individuals were observed in the population. This number of observed *Gyrodactylus* spp. individuals is needed to calculate rarefaction in EstimateS 8.2.0. # None of tests were significant. (DOC 116 kb) Additional file 3:
**Figure of distribution of** ***Gyrodactylus*** **spp. individuals.** Distribution of *Gyrodactylus* spp. individuals in the total dataset of 1278 individual minnows (*Phoxinus phoxinus*) hosts from 43 populations in Norway. Here the 14 native and 23 stocked populations have been pooled. On the left axis is the probability of occurrence and on right axis is given counts. (DOCX 76 kb)
