# Supplementary material for: Gyrodactylus spp. diversity in native and introduced minnow (Phoxinus phoxinus) populations: no support for “the enemy release” hypothesis
Source: Parasit Vectors. 2016 Jan 28;9:51. doi: 10.1186/s13071-016-1306-y (PMC4730603; doi:10.1186/s13071-016-1306-y)
Supplement: Additional file 1: Table S1. — A table of other fish species in the sampled localities. All the Norwegian minnow (Phoxinus phoxinus) populations used in the study and the other fish species in the same locality. The species of fish are: AC = Arctic charr (Salvelinus alpinus) BL = bleak (Alburnus alburnus), BR = bream (Abramis brama), BT = brown trout (Salmo trutta), BU = burbot (Lota lota), CC = crucian carp (Carassius carassius), CH = chub (Leuciscus cephalus), EE = eel (Anguilla anguilla), GR = grayling (Thymallus thymallus), PE = perch (Perca fluviatilis), PI = pike (Esox lucius), RU = ruffe (Gymnocephalus cernuus), SS = Siberian sculpin (Cottus poecilopus), VE = vendace (Coregonus albula), WB = white bream (Blicca bjoerkna), WF = European whitefish, SM = smelt (Osmerus eperlanus), ID = ide (Leuciscus idus), DA = dace (Leuciscus leuciscus), 9-SB = nine-spined stickleback (Pungitius pungitius) and 3-SB = tree-spined stickleback (Gasterosteus aculeatus). (DOC 192 kb) [file 13071_2016_1306_MOESM1_ESM.doc]

Additional file 1

*Gyrodactylus* spp. diversity in native and introduced minnow (*Phoxinus phoxinus*) populations: no support for “the enemy release” hypothesis

Parasite & Vectors

R. A. Pettersen^*^, K. Østbye, J. Holmen, L. A. Vøllestad & T. A. Mo

* Corresponding author, e-mail: [rubenap@ibv.uio.no](mailto:rubenap@ibv.uio.no)

**Additional file 1. Table 1**. All the Norwegian minnow (*Phoxinus phoxinus*) populations used in the study and the other fish species in the same locality. The species of fish are: AC = Arctic charr (*Salvelinus alpinus*) BL = bleak (*Alburnus alburnus*), BR = bream (*Abramis brama*), BT = brown trout (*Salmo trutta*), BU = burbot (*Lota lota*), CC = crucian carp (*Carassius carassius*), CH = chub (*Leuciscus cephalus*), EE = eel (*Anguilla anguilla*), GR = grayling (*Thymallus thymallus*), PE = perch (*Perca fluviatilis*), PI = pike (*Esox lucius*), RU = ruffe (*Gymnocephalus cernuus*), SS = Siberian sculpin (*Cottus poecilopus*), VE = vendace (*Coregonus albula*), WB = white bream (*Blicca bjoerkna*), WF = European whitefish, SM = smelt (*Osmerus eperlanus*), ID = ide (*Leuciscus idus*), DA = dace (*Leuciscus leuciscus*), 9-SB = nine-spined stickleback (*Pungitius pungitius*) 3-SB = tree-spined stickleback (*Gasterosteus aculeatus*).

|  | Location |  |  | |  | |  | | |  |  |  | |  | | |  | | |  | | |  | | |  | | |  | | |  | | | |  | | |  | | |  | | |  | | |  | |  | | |
| --- | --- | --- | --- | --- | --- | --- | --- | --- | --- | --- | --- | --- | --- | --- | --- | --- | --- | --- | --- | --- | --- | --- | --- | --- | --- | --- | --- | --- | --- | --- | --- | --- | --- | --- | --- | --- | --- | --- | --- | --- | --- | --- | --- | --- | --- | --- | --- | --- | --- | --- | --- | --- |
| Native populations | | *AC* | *BL* | *BR* | | *BT* | | | | *BU* | *CC* | *CH* | | | *EE* | | | *GR* | | | *PE* | | | *PI* | | | *RU* | | | *SS* | | | *VE* | | | | *WF* | | | *SM* | | | *ID* | | | *DA* | | *9-SP* | | *3-SP* | |  |
| 1 | Sørkedalselva |  |  |  | | x | | | |  |  |  | | | x | | |  | | | x | | | x | | |  | | |  | | |  | | | |  | | |  | | |  | | |  | | x | | x | |  |
| 2 | Fallselva |  |  |  | | x | | | |  |  |  | | x | | |  | | | x | | | x | | |  | | |  | | |  | | |  | | | |  | | |  | | |  | | | x | | x | | |
| 3 | Hunnselva |  | x | x | | x | | | | x | x | x | | x | | | x | | | x | | | x | | | x | | | x | | | x | | | | x | | | x | | | x | | | x | | | x | | x | | |
| 4 | Elverum |  | x | x | | x | | | | x |  | x | |  | | | x | | | x | | | x | | | x | | | x | | | x | | | | x | | | x | | | x | | | x | | |  | | x | | |
| 5 | Julussa |  |  |  | | x | | | |  |  |  | |  | | | x | | |  | | | x | | |  | | | x | | |  | | | | x | | |  | | |  | | |  | | |  | | x | | |
| 6 | Søre Osa |  |  |  | | x | | | |  |  |  | |  | | | x | | |  | | | x | | |  | | | x | | |  | | | | x | | |  | | |  | | |  | | |  | | x | | |
| 7 | Femunden | x |  |  | | x | | | | x |  |  | |  | | | x | | | x | | | x | | |  | | |  | | |  | | | | x | | |  | | |  | | |  | | |  | |  | | |
| 8 | Sørli | x |  |  | | x | | | |  |  |  | |  | | |  | | |  | | |  | | |  | | |  | | |  | | | |  | | |  | | |  | | |  | | |  | | x | | |
| 9 | Stuorajavri | x |  |  | | x | | | |  |  |  | |  | | |  | | |  | | | x | | |  | | |  | | |  | | | | x | | |  | | |  | | |  | | |  | |  | | |
| 10 | Tana | x |  |  | | x | | | | x |  |  | | x | | | x | | |  | | | x | | |  | | |  | | |  | | | | x | | |  | | |  | | |  | | | x | | x | | |
| 11 | Asdøltjern |  |  |  | | x | | | |  |  |  | |  | | |  | | | x | | |  | | |  | | |  | | |  | | | |  | | |  | | |  | | |  | | |  | | x | | |
| 12 | Sagelva |  |  |  | | x | | | |  |  |  | |  | | |  | | | x | | |  | | |  | | |  | | |  | | | |  | | |  | | |  | | |  | | | x | | x | | |
| 13 | Fiskebekktjern |  |  | |  | | x | |  | |  |  | |  | | |  | | |  | | |  | | |  | | |  | | |  | | | |  | | |  | | |  | | |  | | |  | |  | | |
| 14 | Landsjøen |  |  | |  | | x | |  | |  |  | |  | | |  | | | x | | |  | | |  | | |  | | |  | | | |  | | |  | | |  | | |  | | |  | |  | | |
|  | |  | | | | | |  | |  |  | |  | | |  | | |  | | |  | | |  | | |  | | |  | | |  | | | |  | | |  | | |  | | |  | |  | |  | |
|  | |  | | | | | |  | |  |  | |  | | |  | | |  | | |  | | |  | | |  | | |  | | |  | | | |  | | |  | | |  | | |  | |  | |  | |
| Introduced populations | | *AC* | *BL* | | *BR* | | *BT* | | | *BU* | *CC* | *CH* | | *EE* | | | *GR* | | | *PE* | | | *PI* | | | *RU* | | | *SS* | | | *VE* | | | | *WF* | | | *SM* | | | *ID* | | | *DA* | | | *9-SP* | | *3-SP* | | |
| 15 | Øteren |  |  | |  | | x | | |  |  |  | |  | | |  | | |  | | |  | | |  | | |  | | |  | | | |  | | |  | | |  | | |  | | |  | |  | | |
| 16 | Strandavatn |  |  | |  | | x | | |  |  |  | |  | | |  | | |  | | |  | | |  | | |  | | |  | | | |  | | |  | | |  | | |  | | |  | |  | | |
| 15 | Stolsvatnet |  |  | |  | | x | | |  |  |  | |  | | |  | | |  | | |  | | |  | | |  | | |  | | | |  | | |  | | |  | | |  | | |  | |  | | |
| 18 | Hustjern |  |  | |  | | x | | |  |  |  | |  | | |  | | |  | | |  | | |  | | |  | | |  | | | |  | | |  | | |  | | |  | | |  | |  | | |
| 19 | Hallingsdalselva |  |  | |  | | x | | |  |  |  | |  | | |  | | |  | | |  | | |  | | |  | | |  | | | |  | | |  | | |  | | |  | | |  | |  | | |
| 20 | Tisleia |  |  | |  | | x | | |  |  |  | |  | | |  | | |  | | |  | | |  | | |  | | |  | | | |  | | |  | | |  | | |  | | |  | |  | | |
| 21 | Bygdin |  |  | |  | | x | | |  |  |  | |  | | |  | | |  | | |  | | |  | | |  | | |  | | | |  | | |  | | |  | | |  | | |  | |  | | |
| 22 | Vinstri |  |  | |  | | x | | |  |  |  | |  | | |  | | |  | | |  | | |  | | |  | | |  | | | | x | | |  | | |  | | |  | | |  | |  | | |
| 23 | Vinstervatna Ø. |  |  | |  | | x | | |  |  |  | |  | | |  | | |  | | |  | | |  | | |  | | |  | | | | x | | |  | | |  | | |  | | |  | |  | | |
| 24 | Birisjøen |  |  | |  | | x | | |  |  |  | |  | | |  | | |  | | |  | | |  | | |  | | |  | | | |  | | |  | | |  | | |  | | |  | |  | | |
| 25 | Otta |  |  | |  | | x | | |  |  |  | |  | | | x | | |  | | |  | | |  | | |  | | |  | | | |  | | |  | | |  | | |  | | |  | |  | | |
| 26 | Mjåvatn |  |  | |  | | x | | |  |  |  | |  | | |  | | |  | | |  | | |  | | |  | | |  | | | |  | | |  | | |  | | |  | | |  | |  | | |
| 27 | Totak | x |  | |  | | x | | |  |  |  | |  | | |  | | |  | | |  | | |  | | |  | | |  | | | |  | | |  | | |  | | |  | | |  | |  | | |
| 28 | Møsvatn | x |  | |  | | x | | |  |  |  | |  | | |  | | |  | | |  | | |  | | |  | | |  | | | |  | | |  | | |  | | |  | | |  | |  | | |
| 20 | Follsjå |  |  | |  | | x | | |  |  |  | |  | | |  | | |  | | |  | | |  | | |  | | |  | | | |  | | |  | | |  | | |  | | |  | |  | | |
| 30 | Stigstuv |  |  | |  | | x | | |  |  |  | |  | | |  | | |  | | |  | | |  | | |  | | |  | | | |  | | |  | | |  | | |  | | |  | |  | | |
| 31 | Lægreid |  |  | |  | | x | | |  |  |  | |  | | |  | | |  | | |  | | |  | | |  | | |  | | | |  | | |  | | |  | | |  | | |  | |  | | |
| 32 | Tunhovd | x |  | |  | | x | | |  |  |  | |  | | |  | | |  | | |  | | |  | | |  | | |  | | | |  | | |  | | |  | | |  | | |  | |  | | |
| 33 | Kippesjøen |  |  | |  | | x | | |  |  |  | |  | | |  | | | x | | |  | | |  | | |  | | |  | | | |  | | |  | | |  | | |  | | |  | |  | | |
| 34 | Heggefjorden |  |  | |  | | x | | |  |  |  | |  | | |  | | |  | | |  | | |  | | |  | | |  | | | |  | | |  | | |  | | |  | | |  | |  | | |
| 35 | Vinstervanna V |  |  | |  | | x | | |  |  |  | |  | | |  | | |  | | |  | | |  | | |  | | |  | | | |  | | |  | | |  | | |  | | |  | |  | | |
| 36 | Grovi |  |  | |  | | x | | |  |  |  | |  | | |  | | |  | | |  | | |  | | |  | | |  | | | |  | | |  | | |  | | |  | | |  | |  | | |
| 37 | Jølstervatn |  |  | |  | | x | | |  |  |  | |  | | |  | | |  | | |  | | |  | | |  | | |  | | | |  | | |  | | |  | | |  | | |  | |  | | |
| 38 | Lesjaskogsvatn |  |  | |  | | x | | |  |  |  | |  | | | x | | |  | | |  | | |  | | |  | | |  | | | |  | | |  | | |  | | |  | | |  | |  | | |
| 39 | Glasåtjern |  |  | |  | | x | | |  |  |  | |  | | |  | | |  | | | x | | |  | | |  | | |  | | | |  | | |  | | |  | | |  | | |  | |  | | |
| 40 | Essandsjøen | x |  | |  | | x | | | x |  |  | |  | | |  | | |  | | |  | | |  | | |  | | |  | | | |  | | |  | | |  | | |  | | |  | |  | | |
| 41 | Risvatnet |  |  | |  | | x | | |  |  |  | | x | | |  | | |  | | |  | | |  | | |  | | |  | | | |  | | |  | | |  | | |  | | |  | |  | | |
| 42 | Limingen | x |  | |  | | x | | |  |  |  | |  | | |  | | |  | | |  | | |  | | |  | | |  | | | |  | | |  | | |  | | |  | | |  | |  | | |
| 43 | Store Majavatn | x |  | |  | | x | | |  |  |  | |  | | |  | | |  | | |  | | |  | | |  | | |  | | | |  | | |  | | |  | | |  | | |  | |  | | |
