# Supplementary material for: Gyrodactylus spp. diversity in native and introduced minnow (Phoxinus phoxinus) populations: no support for “the enemy release” hypothesis
Source: Parasit Vectors. 2016 Jan 28;9:51. doi: 10.1186/s13071-016-1306-y (PMC4730603; doi:10.1186/s13071-016-1306-y)
Supplement: Additional file 2: — A table of tests result of demographic bottleneck. All the Norwegian minnow (Phoxinus phoxinus) populations used in the study. Information from tests of demographic bottleneck using Bottleneck 1.2.0.2. (Piry et al. 1999) with the two-phase model (TPM) and the Wilcoxon sign-rank test, and demographic expansion (where the intra-locus k-test identify signals of recent population expansion, while the inter-locus g-test identify signals of more ancient population expansion) using Kg-test (Reich et al. 1999). Footnotes: *Samples used in the rarefaction analysis using EstimateS 8.2.0 (Colwell 2011) where more than 5 Gyrodactylus spp. individuals were observed in the population. This number of observed Gyrodactylus spp. individuals is needed to calculate rarefaction in EstimateS 8.2.0. # None of tests were significant. (DOC 116 kb) [file 13071_2016_1306_MOESM2_ESM.doc]

Additional file 2

# *Gyrodactylus* spp. diversity in native and introduced minnow (*Phoxinus phoxinus*) populations: no support for “the enemy release” hypothesis

Parasite & Vectors

Ruben A Pettersen^*^, Kjartan Østbye, Johannes Holmen, Leif A Vøllestad, Tor A Mo

* Corresponding author, e-mail: rubenap@ibv.uio.no

**Additional file 2 -** **A table of tests result of demographic bottleneck**

All the Norwegian minnow (*Phoxinus phoxinus*) populations used in the study. Information from tests of demographic bottleneck using Bottleneck 1.2.0.2. [57] with the two-phase model (TPM) and the Wilcoxon sign-rank test, and demographic expansion (where the intra-locus k-test identify signals of recent population expansion, while the inter-locus g-test identify signals of more ancient population expansion) using Kg-test [60].

| 12 | Location | Number  *P. phoxinus* | | *Wilcoxon*  *p-value#* | *K-test*  *p-value#* | *g-test test*  *statistic#* |
| --- | --- | --- | --- | --- | --- | --- |
| Native populations | |  | |  |  |  |
| 1* | Sørkedalselva | 55 | | 0.326 | 0.992 | 0.527 |
| 2* | Fallselva | 41 | | 0.326 | 0.866 | 0.739 |
| 3* | Hunnselva | 45 | | 0.422 | 0.992 | 1.328 |
| 4* | Elverum | 18 | | 0.285 | 0.866 | 0.774 |
| 5* | Julussa | 10 | | 0.326 | 0.999 | 0.903 |
| 6* | Søre Osa | 20 | | 0.674 | 0.992 | 0.567 |
| 7* | Femunden | 22 | | 0.589 | 0.690 | 0.911 |
| 8* | Sørli | 19 | | 0.958 | 1.000 | 1.519 |
| 9* | Stuorajavri | 21 | | 0.500 | 0.959 | 0.623 |
| 10* | Tana | 25 | | 0.215 | 0.866 | 0.767 |
| 11 | Asdøltjern | 40 | | 0.981 | 0.999 | 0.540 |
| 12 | Sagelva | 54 | | 0.787 | 0.992 | 0.852 |
| 13 | Fiskebekktjern | 20 | | 0.589 | 0.886 | 0.591 |
| 14 | Landsjøen | 17 | | 0.545 | 0.999 | 0.339 |
|  | |  |  | |  |  |
| Introduced populations | |  | |  |  |  |
| 15* | Øteren | 20 | | 0.179 | 0.992 | 0.551 |
| 16* | Strandavatn | 33 | | 0.714 | 0.999 | 0.486 |
| 17* | Stolsvatnet | 60 | | 0.687 | 0.959 | 0.385 |
| 18* | Hustjern | 16 | | 0.125 | 0.999 | 1.244 |
| 19* | Hallingsdalselva | 22 | | 0.490 | 0.959 | 1.295 |
| 20* | Tisleia | 40 | | 0.947 | 0.992 | 0.856 |
| 21* | Bygdin | 36 | | 0.473 | 0.992 | 0.717 |
| 22* | Vinstri | 28 | | 0.278 | 0.992 | 0.448 |
| 23* | Vinstervatna Ø. | 122 | | 0.312 | 0.992 | 1.124 |
| 24* | Birisjøen | 21 | | 0.001 | 1.000 | 1.046 |
| 25* | Otta | 8 | | 0.004 | 0.992 | 0.892 |
| 26 | Mjåvatn | 18 | | 0.455 | 0.459 | 2.515 |
| 27 | Totak | 20 | | 0.303 | 0.866 | 1.311 |
| 28 | Møsvatn | 17 | | 0.570 | 0.992 | 0.305 |
| 20 | Follsjå | 15 | | 0.843 | 0.992 | 0.618 |
| 30 | Stigstuv | 52 | | 0.632 | 0.241 | 2.877 |
| 31 | Lægreid | 54 | | 0.097 | 0.959 | 0.490 |
| 32 | Tunhovd | 33 | | 0.714 | 0.992 | 1.012 |
| 33 | Kippesjøen | 17 | | 0.987 | 1.000 | 1.390 |
| 34 | Heggefjorden | 25 | | 0.460 | 0.999 | 0.666 |
| 35 | Vinstervanna V | 44 | | 0.318 | 0.992 | 0.593 |
| 36 | Grovi | 20 | | 0.273 | 0.866 | 1.210 |
| 37 | Jølstervatn | 38 | | 0.634 | 0.999 | 1.084 |
| 38 | Lesjaskogsvatn | 16 | | 0.008 | 0.992 | 0.568 |
| 39 | Glasåtjern | 10 | | 0.674 | 0.992 | 0.618 |
| 40 | Essandsjøen | 30 | | 0.578 | 0.992 | 1.110 |
| 41 | Risvatnet | 20 | | 0.234 | 1.000 | 1.090 |
| 42 | Limingen | 20 | | 0.285 | 0.999 | 1.444 |
| 43 | Store Majavatn | 16 | | 0.320 | 0.999 | 1.570 |

* Samples used in the rarefaction analysis using EstimateS 8.2.0 [ where more than 5 *Gyrodactylus* spp. individuals were observed in the population. This number of observed *Gyrodactylus* spp. individuals is needed to calculate rarefaction in EstimateS 8.2.0.

# None of tests were significant.

References

57. Piry S, Luikart G, Cornuet JM: **BOTTLENECK: A computer program for detecting recent reductions in the effective population size using allele frequency data**. *J Hered* 1999, **90**(4):502-503.

60. Reich DE, Goldstein DB: **Genetic evidence for a Paleolithic human population expansion in Africa**. *Proc Natl Acad Sci U S A* 1998, **95**(14):8119-8123.

61. Colwell R, K.: **EstimateS 8.2.0: Statistical estimation of species richness and shared species from samples**. *User’s Guide and application published at:* [*http://purloclcorg/estimates*](http://purloclcorg/estimates)2011.
