# Supplementary material for: Gyrodactylus spp. diversity in native and introduced minnow (Phoxinus phoxinus) populations: no support for “the enemy release” hypothesis
Source: Parasit Vectors. 2016 Jan 28;9:51. doi: 10.1186/s13071-016-1306-y (PMC4730603; doi:10.1186/s13071-016-1306-y)
Supplement: Additional file 3: — Figure of distribution of Gyrodactylus spp. individuals. Distribution of Gyrodactylus spp. individuals in the total dataset of 1278 individual minnows (Phoxinus phoxinus) hosts from 43 populations in Norway. Here the 14 native and 23 stocked populations have been pooled. On the left axis is the probability of occurrence and on right axis is given counts. (DOCX 76 kb) [file 13071_2016_1306_MOESM3_ESM.docx]

Additional file 3 Parasite & Vektors

*Gyrodactylus* spp. diversity in native and introduced minnow (*Phoxinus phoxinus*) populations: no support for “the enemy release” hypothesis.

Ruben A Pettersen^*^, Kjartan Østbye, Johannes Holmen, Leif A Vøllestad, Tor A Mo

* Corresponding author, e-mail: rubenap@ibv.uio.no

### Additional file 3 – Figure of distribution of *Gyrodactylus* spp. individuals


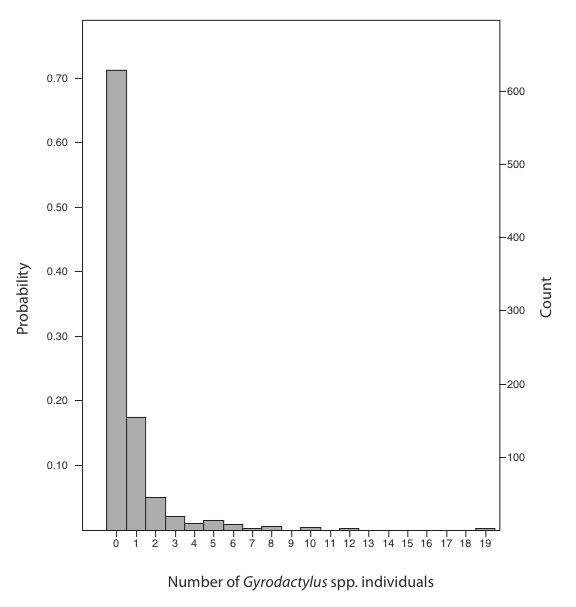


**Additional file 3**. **Figure 1**. Distribution of *Gyrodactylus* spp. individuals in the total dataset of 1278 individual minnows (*Phoxinus phoxinus*) hosts from 43 population in Norway. Here the 14 native and 23 stocked populations have been pooled. On the left axis is the probability of occurrence and on right axis is given counts.
